# Effective inactivated influenza vaccine for the elderly using a single-stranded RNA-based adjuvant

**DOI:** 10.1038/s41598-021-91445-3

**Published:** 2021-06-07

**Authors:** Yoo-Jin Bang, So-Hee Hong, Hyo-Jung Park, Hye Won Kwak, Yu-Sun Lee, Jae-Yong Kim, Hyeong-Jun Park, Seo-Hyeon Bae, Hye-Jung Kim, Yun-Hee Kim, Hae Li Ko, Sang-In Park, Hun Kim, Gyeongjoo Park, Man-Seong Park, Jun Chang, Jae-Hwan Nam

**Affiliations:** 1 Department of Medical and Biological Sciences, The Catholic University of Korea, Bucheon, Gyeonggi-do Republic of Korea; 2 BK Plus Department of Biotechnology, The Catholic University of Korea, Bucheon, Gyeonggi-do Republic of Korea; 3 Department of R&D, SK Bioscience, Pangyoro, Bundang-gu, Republic of Korea; 4 Scripps Korea Antibody Institute, Chuncheon, Kangwon-do Republic of Korea; 5 Graduate School of Pharmaceutical Sciences, Ewha Womans University, Seoul, Republic of Korea; 6 Department of Microbiology, College of Medicine, Korea University, Seoul, Republic of Korea

**Keywords:** Immunology, Vaccines, Infectious diseases

## Abstract

There is an unmet need for new influenza vaccine strategies that compensate for impaired vaccine responses in elderly individuals. Here, we evaluated the effectiveness of a single-stranded RNA (ssRNA) as an adjuvant to enhance the efficacy of inactivated influenza vaccine (IIV) in mouse models. Immunization with the ssRNA along with IIV reduced viral titers as well as pathological and inflammatory scores in the lungs after influenza challenge in aged mice. ssRNA induced balanced Th1/Th2 responses with an increase in IgA titers. Moreover, the ssRNA adjuvant markedly increased the frequency of influenza HA-specific T cells and IFN-γ production along with the expression of genes related to innate and adaptive immune systems that could overcome immunosenescence in aged mice. Our findings indicate that ssRNA is an efficient vaccine adjuvant that boosts cellular and humoral immunity in aged mice, demonstrating its potential as a novel adjuvant for currently available influenza virus vaccines for elderly individuals.

## Introduction

Influenza caused by influenza viruses A and B is a common infectious respiratory disease occurring in the human population. According to the World Health Organization (WHO), influenza virus infects 2–10% of the world’s population and causes 250,000–500,000 deaths annually^1,2^. Similar to the cases observed in other respiratory diseases, immunocompromised individuals, such as the elderly, children, and those with other chronic illnesses, are particularly vulnerable to influenza-associated mortality. It was reported that approximately 70–85% of the deaths due to flu and 50–70% of the flu-associated hospitalization cases occurred in patients older than 65 years^3^. Therefore, influenza is not a negligible disease in the elderly.

Although vaccination is the most efficacious method to prevent the development of infectious diseases, the responsiveness of influenza vaccine is markedly reduced in elderly individuals owing to the occurrence of immunological aging^4,5^. One of the major characteristics of immunological aging is the occurrence of immunosenescence, a complex process that affects both innate and adaptive immune responses. In addition to decreased numbers of circulating monocytes and dendritic cells, reduced phagocytic activity and impaired antigen presentation are associated with aging. T and B cells are also markedly affected by aging^6,7^. The most significant change within the aging T cell population is the contraction of naïve T cells due to thymic atrophy. Additionally, impaired T cell activation, effector T cell failure, and long-lived memory T cell generation have been associated with aging^8,9^. These defects in T cells have been associated with decreases in co-stimulatory molecule expression and cytokine production. A similar phenomenon is also observed in B cells. Decreased generation of naïve B cells from the bone marrow results in the contraction of B cell repertoires^10^. Consequently, this reduction restricts the number of B cell clones that can respond to new antigens^11^. Moreover, aged memory B cells exhibit reduced ability to differentiate into antibody-secreting cells as well as decreased antigen-specific antibody production and germinal center formation^12^.

Another important immunological feature that accompanies aging is persistent, sterile, and low-grade inflammation, also called inflammaging^13^. Higher levels of several pro-inflammatory cytokines, chemokines, and C-reactive protein were detected both within the tissue microenvironments and in blood of aged individuals^13^. This phenomenon is regarded as an obstacle for the induction of proper immune responses to pathogens or vaccines because it impedes the organism’s ability to recognize stimuli. Thus, aged individuals may need higher threshold levels to activate immune cells than that of young individuals^14,15^.

An adjuvant is a substance that improves the immunogenicity of vaccines. Although a few adjuvants, such as aluminum salt, MF59, and AS03, are currently used in influenza vaccines to enhance immune responses, they do not induce sufficient Th1/2 responses^16,17^. Moreover, their efficacy is suboptimal, especially in the elderly. Only 30–40% of individuals over 65 years of age experience influenza vaccine-induced immunogenicity, despite the vaccine and circulating virus being antigenically matched^5,18^. In contrast, the efficacy of vaccine ranges between 70 and 90% in individuals under 65 years. Therefore, it is of utmost interest to explore a new influenza vaccine strategy that induces protective responses and overcomes immunological aging.

We previously developed a novel single-stranded RNA (ssRNA) platform that originated from cricket paralysis virus (CrPV) internal ribosome entry sites (IRES). We showed that the ssRNA could act as an effective adjuvant when formulated with an inactivated influenza vaccine (IIV) or MERS-CoV spike protein vaccine in young mice^19,20^. Moreover, the ssRNA adjuvant bestowed cross-protection against heterologous influenza virus^20^. In the present study, we extended our previous research and tested whether an ssRNA adjuvant could potentially enhance the efficacy of IIV in elderly through conduction of experiments using aged mice.

## Results

### IIV formulated with ssRNA induces a balanced IgG1/IgG2a immune response and elicits the production of higher neutralizing antibody titers in young and aged mice

**Figure 1:**
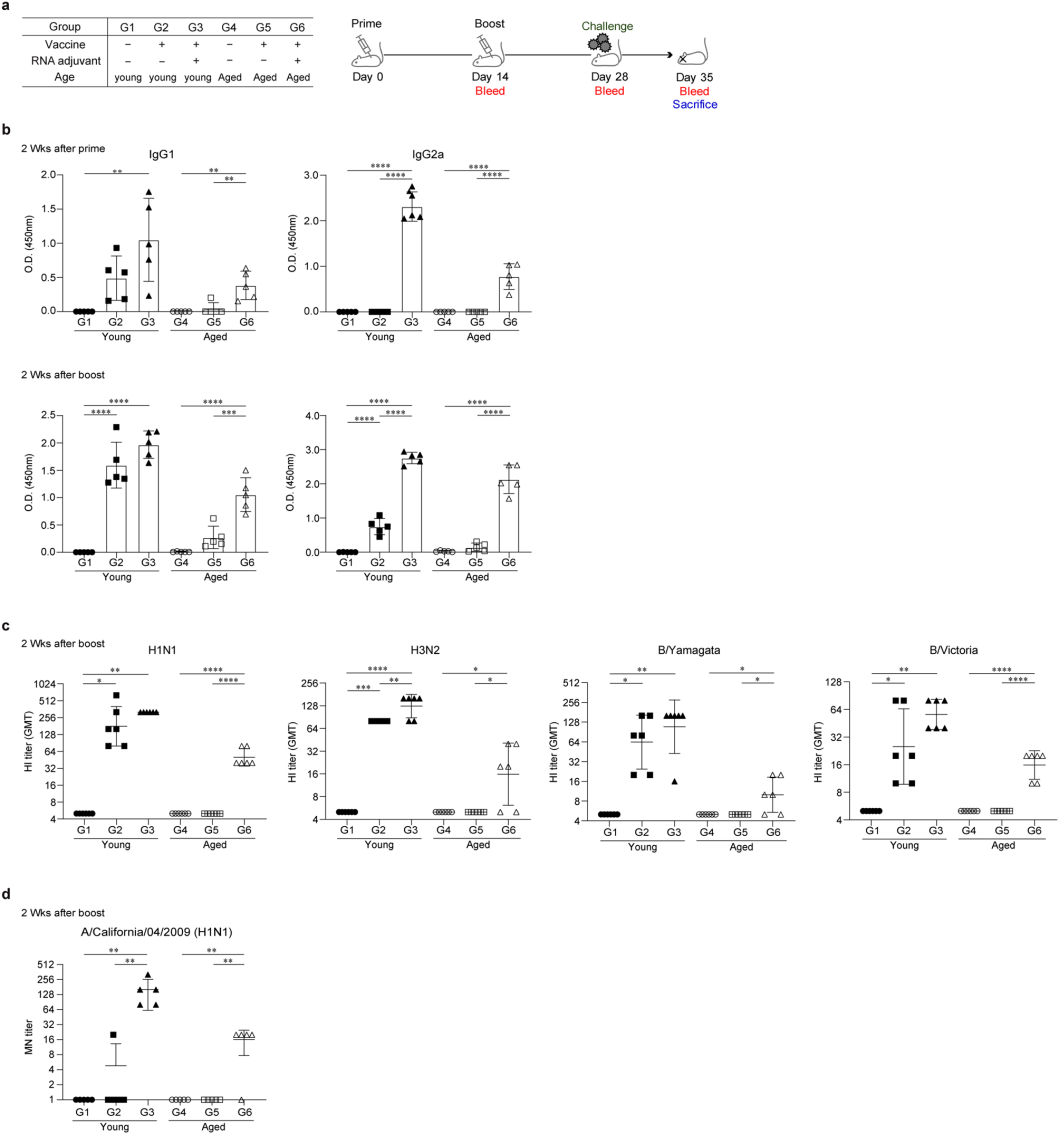
Inactivated influenza vaccine (IIV) formulated with ssRNA augments the IIV-induced humoral responses. BALB/c mice were intramuscularly immunized with 0.6 μg IIV with or without ssRNA adjuvant at an interval of 2 weeks. 2 weeks after the boost schedule, mice were challenged with A/California/04/2009 virus. To perform IgG, IgG2a, hemagglutination inhibition, and microneutralization assays, sera were collected at 2 weeks after the prime and boost schedule. Data are represented as mean ± SD. The data were statistically analyzed by one-way ANOVA. The significance of differences between groups are indicated by bars and symbols as follows: *p < 0.05; **p < 0.01; ***p < 0.001; ****p < 0.0001. (**a**) Immunization groups and schedule for immunization and challenge. n = 5 mice for G1 to G6. (**b**) IIV-specific IgG1 and IgG2a levels measured by ELISA 2 weeks after the prime and boost schedule. n = 5 mice for G1–G6. (**c**) Hemagglutination inhibition titer against vaccine strains measured by hemagglutination inhibition assay 2 weeks after the boost schedule. Data represent geomean of duplicated results. n = 3 mice for G1–G6. (**d**) Microneutralization titer against A/California/04/2009 measured by microneutralization assay 2 weeks after the boost schedule. n = 5 mice for G1–G6.

To assess the adjuvant effect of ssRNA on humoral responses, young (6-week-old) and aged (21-month-old) mice were subjected to intramuscular vaccination with IIV formulated with or without ssRNA (20 μg), and in a prime/boost schedule, their blood samples were collected as described in Fig. 1a. Interestingly, ssRNA adjuvant considerably increased the humoral response in both young and aged mice. IIV alone induced substantial levels of IgG1 (average O.D. value: 1.6 ± 0.4) and IgG2a (O.D. value: 0.75 ± 0.2) in young mice (G2), but it rarely induced the production of IgG1 (O.D. value: 0.3 ± 0.2) and IgG2a (OD value: 0.15 ± 0.12) antibodies in aged mice (G5), even 2 weeks after the boost schedule (Fig. 1b). However, IIV formulated with ssRNA-immunized aged mice (G6) showed increases in IgG1 (O.D. value: 0.38 ± 0.21) and IgG2a (OD value: 0.78 ± 0.28) antibodies 2 weeks after the prime schedule (Fig. 1b), and significant increases in IgG1 (O.D. value: 1.1 ± 0.31) and IgG2a (O.D. value: 2.1 ± 0.42) antibodies 2 weeks after the boost schedule. A similar pattern was observed 1 week after the boost schedule (Supplementary Fig. S1). Notably, ssRNA adjuvant remarkably induced IgG2a antibody production. In young mice, IIV alone (G2) could not induce IgG2a antibodies after the prime schedule; however, they were detected in IIV formulated ssRNA-immunized mice (G3) after the prime schedule. In aged mice, considerable levels of serum IgG2a were detected only in the IIV formulated with ssRNA-immunized groups (G6) at all observation time points (Fig. 1b).

Furthermore, A considerable increase in hemagglutination inhibition (HI) titers against vaccine strains A/Michigan/45/2015 (NYMC X-275) for A/H1N1, A/Singapore/INFIMH-16-0019/2016 (IVR-186) for A/H3N2, B/Phuket/3073/2013 for B/Yamagata, B/Maryland/15/2016 for B/Victoria, and A/California/04/2009 (H1N1), the surrogate model for A/Michigan/45/2015 (NYMC X-275), for H1N1 was detected in the IIV formulated with ssRNA-immunized groups. Furthermore, microneutralization antibody titers against A/California/04/2009 (H1N1) were detected in the IIV formulated with ssRNA-immunized groups in both young and aged groups (G3 and G6) (Fig. 1c,d, Supplementary Fig. S2).

### IIV formulated with ssRNA restricts viral replication and reduces influenza-induced pathogenesis in young and aged mice

**Figure 2:**
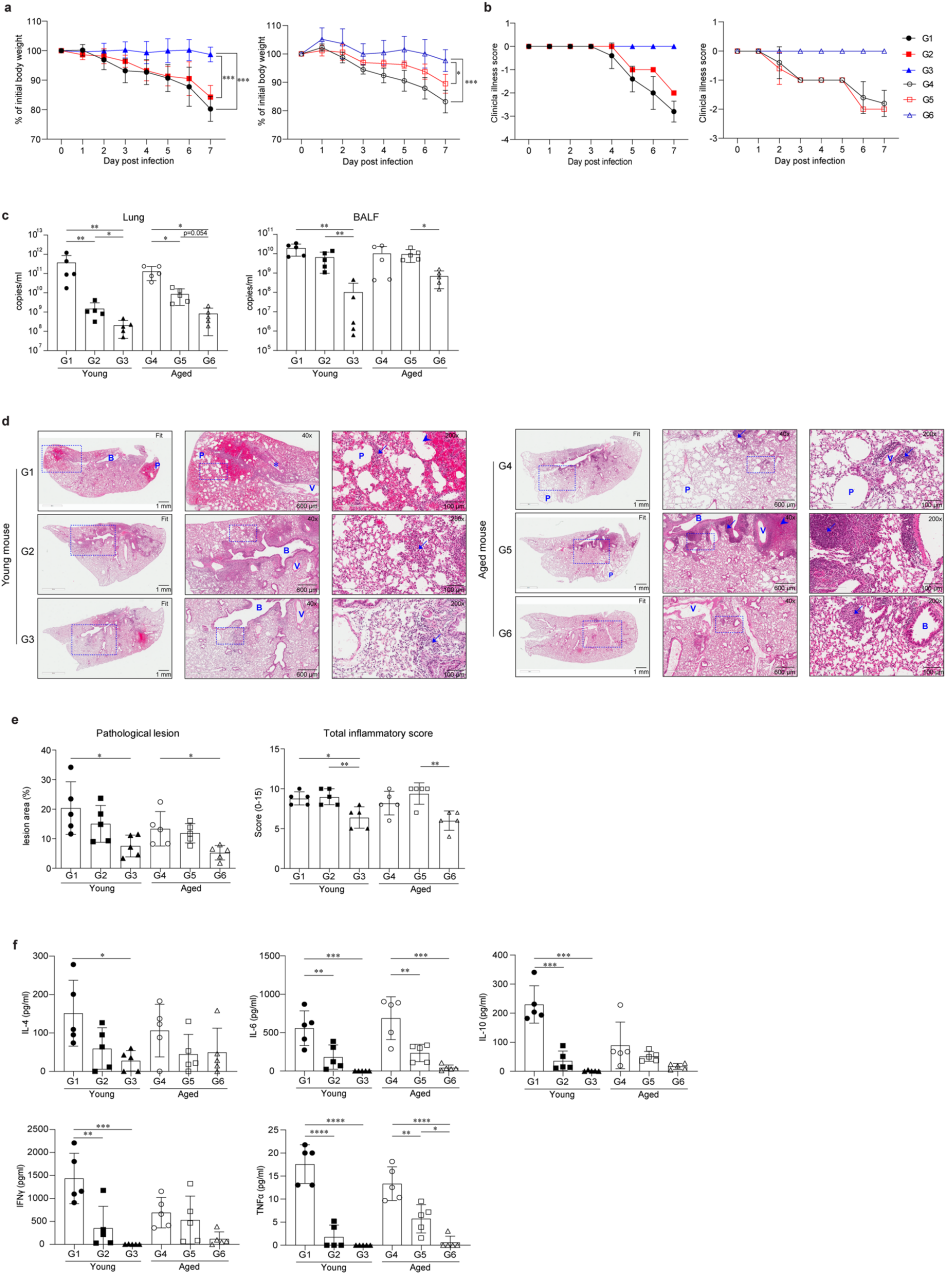
Inactivated influenza vaccine (IIV) formulated with ssRNA reduces viral replication and pathogenesis and induces protection against influenza virus. BALB/c mice were challenged with A/California/04/2009 virus 2 weeks after the boost schedule and sacrificed after 1 week as described in Fig. 1a. The lungs were harvested 1 week after the virus challenge, fixed, processed, and H&E-stained for lung histopathological evaluation. BALF was harvested 1 week after the challenge. The experimental groups are described in Fig. 1a. Data are represented as mean ± SD and statistically analyzed by one-way ANOVA and Student’s *t*-test. The significance of differences between groups are indicated by bars and symbols as follows: *p < 0.05; **p < 0.01; ***p < 0.001; ****p < 0.0001. n = 5 mice for G1 to G6. Percentages of young and aged mice body weight (**a**) and clinical illness score (**b**) studied for 1 week after challenge with A/California/04/2009. Clinical illness score detailed in “Materials and methods”. (**c**) Viral titer in the lungs and BALF of young and aged mice measured by real-time PCR 1 week after challenge with A/California/04/2009. (**d**) Lungs were stained with hematoxylin and eosin (H&E). B, bronchus or bronchi; V, blood vessel; P, pulmonary emphysema; arrow, inflammatory cells (neutrophils and lymphocytes); arrowhead, hemorrhage; asterisk, exudate. **(e**) Percentages of pathological lesion area and total inflammatory score. (**f**) IL-4, IL-6, IL-10, IFN-γ, and TNF-α levels in BALF measured using the Magnetic Luminex Screening Assay Kit.

Subsequently, we ascertained whether the ssRNA adjuvant application could enhance the protective effects of IIV and reduce virus-induced pathogenesis after viral challenge. As shown in Fig. 2a,b, IIV-immunized groups (G2 and G5) showed slightly delayed or alleviated weight loss and development of clinical illness in both young and aged mice compared to the respective control groups (G1 and G4). In contrast, IIV formulated with ssRNA-immunized groups (G3 and G6) did not show any significant weight loss or development of clinical illness after the challenge performed in young and aged mice (Fig. 2a,b). In line with these results, viral titers in the lungs and bronchoalveolar lavage fluid (BALF) collected 1 week after conduction of the challenge were considerably reduced in groups immunized with IIV formulated with ssRNA (G3 and G6). Average virus titer (copy number) in the lungs of IIV-immunized young or aged mice were 2 × 10^9^ and 9 × 10^9^ copies/mL, respectively; furthermore, these numbers were reduced to 2 × 10^8^ (young) and 8 × 10^8^ (aged) copies/mL in the groups immunized with IIV formulated with ssRNA (G3 and G6) (Fig. 2c).

In addition, the recruitment of inflammatory cells into the lungs (bronchi or blood vessel), parenchymal and vascular inflammation, and bronchiolitis were markedly reduced in groups immunized with IIV formulated with ssRNA (G3 and G6) (Fig. 2d,e). Finally, remarkable reductions in levels of cytokines, such as interleukin (IL)-4, IL-6, IL-10, interferon (IFN)-γ, and tumor necrosis factor (TNF)-α were observed in the BALF of virus-challenged G3 and G6 groups (Fig. 2f).

### IIV formulated with ssRNA increases the generation of influenza-specific T cells and humoral response after viral challenge in young and aged mice

**Figure 3:**
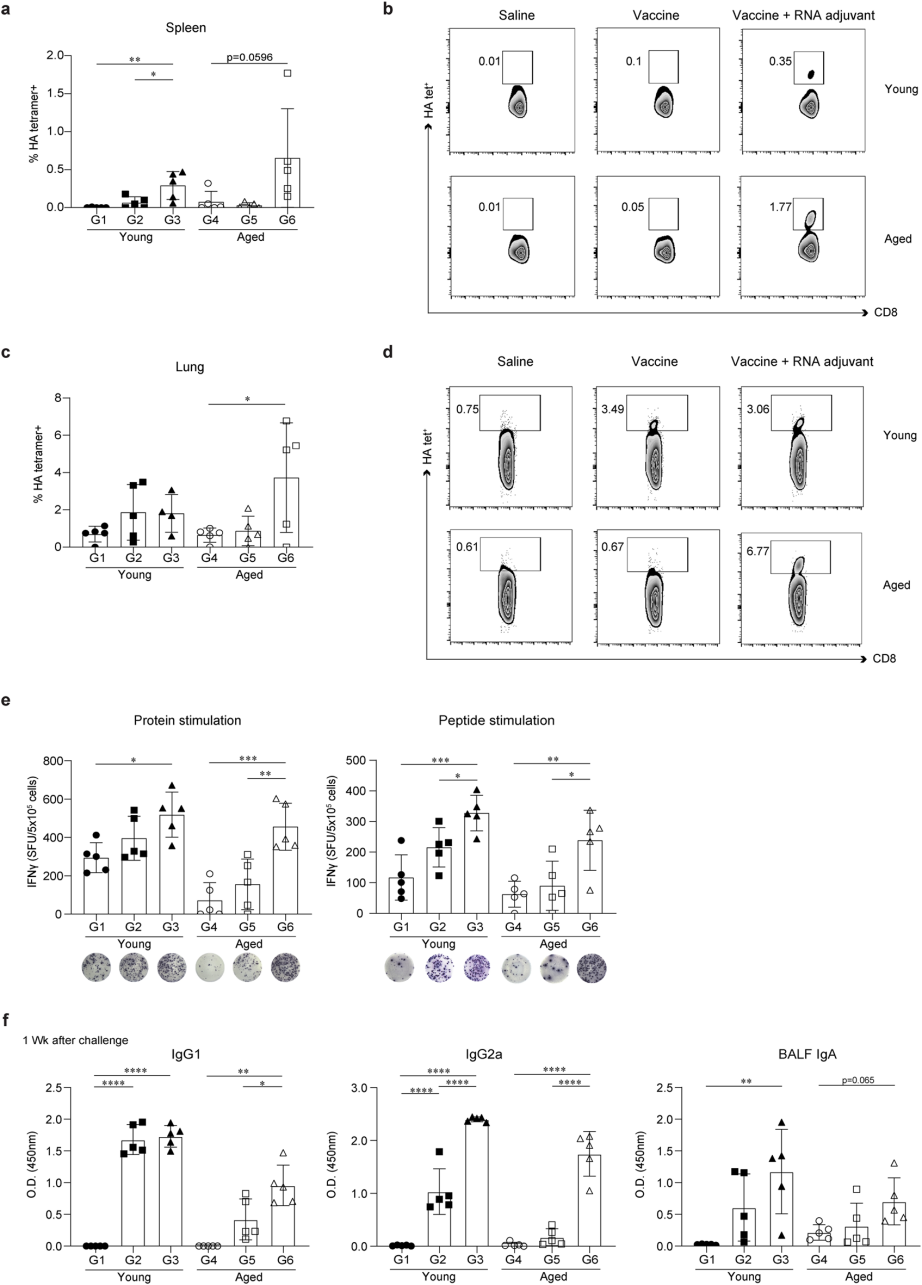
Inactivated influenza vaccine (IIV) formulated with ssRNA increases the generation of influenza HA-specific T cells and humoral response against influenza virus. Splenocytes and lung lymphocytes were harvested 1 week after challenge. For conducting the ELISPOT assay, cells were stimulated for 2 days with or without IIV or influenza HA-specific T cell epitope peptide. Detailed peptide sequences are listed in the “Materials and methods”. For estimation of levels of IgG1, IgG2a, and IgA, sera and BALF were collected 1 week after challenge. The experimental groups are described in Fig. 1a. Data are represented as mean ± SD and were statistically analyzed using the one-way ANOVA. The significance of differences between groups are indicated by bars and symbols as follows: *p < 0.05; **p < 0.01; ***p < 0.001; ****p < 0.0001. Percentages of HA-specific tetramer in the spleen (**a**,**b**) (n = 5 mice for G1 to G6) and lungs (**c**,**d**) (n = 4 mice for G3 and n = 5 mice for G1, G2, G4, G5, and G6) measured by FACS. (**e**) Spots of influenza HA-specific IFN-γ-producing cells in splenocytes measured using ELISPOT. n = 5 mice for G1–G6. (**f**) IIV-specific IgG1, IgG2a, and BALF IgA levels measured using ELISA 1 week after the challenge. n = 5 mice for G1–G6.

As the protection against influenza is also associated with the generation of cytotoxic T cell responses as well as antibody responses, we determined whether the alleviation of influenza symptoms caused by the application of the ssRNA adjuvant was associated with an increase in antigen-specific CD8^+^ T cells. As indicated in Fig. 3a,b, the percentages of HA_533_-specific CD8^+^ T cells in the spleen were markedly increased by the application of the ssRNA adjuvant, in both in young and aged mice 1 week after the challenge. The ssRNA adjuvant did not markedly increase the percentages of HA_533_-specific CD8^+^ T cells in the lungs of young mice, but such an increase was observed in old mice (Fig. 3c,d). Furthermore, the application of the ssRNA adjuvants increased the number of IFN-γ-producing T cells triggered by hemagglutinin (HA) protein and HA peptide in the spleen of young and aged mice (Fig. 3e). Thus, these results indicate that the enhanced protective effects of the ssRNA adjuvant are associated with the generation of T cell-specific immune responses.

ssRNA also increased the serum levels of IgG2a and BALF IgA in young mice after challenge (G3), and more significant increases in the serum levels of IgG1, IgG2a, and BALF IgA were observed in aged mice immunized with IIV formulated with ssRNA (G6) (Fig. 3f).

### ssRNA increases the generation of dendritic cells in the spleen and promotes T cell activation in young and aged mice

**Figure 4:**
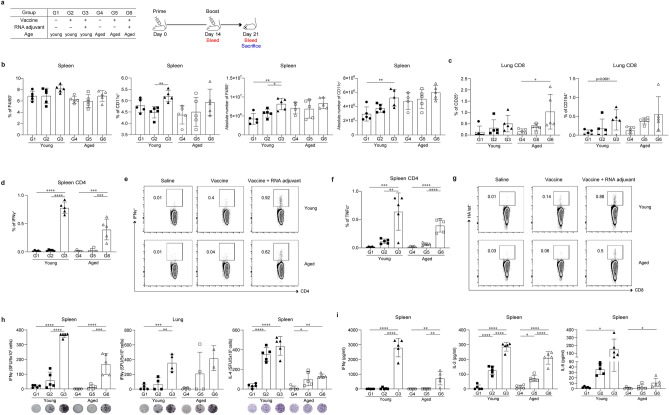
Inactivated influenza vaccine (IIV) formulated with ssRNA increases antigen-presenting cells, activation markers, and cytokine production in T cells. BALB/c mice were intramuscularly immunized with 0.6 μg of IIV with or without ssRNA adjuvant at an interval of 2 weeks. Splenocytes and lung lymphocytes were harvested 1 week after the boost schedule. To detect cytokines, cells were stimulated for 16 h for FACS analysis, for 2 days for ELISPOT assays, and for 3 days for conducting ELISA with or without IIV. Data are represented as mean ± SD and were statistically analyzed by one-way ANOVA. The significance of differences between groups are indicated by bars and symbols as follows: *p < 0.05; **p < 0.01; ***p < 0.001; ***﻿*p < 0.0001. n = 5 mice for G1, G2, G3, G5 and G6. n = 4 or 5 mice for G4. (**a**) Immunization groups and schedule. (**b**) Percentages and absolute number of macrophage and dendritic cells in young and aged mice splenocytes. (**c**) Percentages of CD25 and CD154 in young and aged mice lung lymphocytes. Percentages of (**d**,**e**) IFN-γ and (**f**,**g**) TNF-α in young and aged mice splenocytes. (**h**) Spots of influenza HA-specific IFN-γ- and IL-4-producing cells among splenocytes and lung lymphocytes measured by ELISPOT. (**i**) Concentration of influenza HA-specific IFN-γ, IL-2, and IL-6 in splenocyte culture supernatant measured by ELISA.

To decipher the underlying mechanism of the boosting effects of the ssRNA adjuvants, splenocytes and lung lymphocytes were isolated 1 week after the boost schedule from young and aged mice vaccinated with IIV formulated with or without ssRNA in a prime/boost schedule as described in Fig. 4a. As indicated in Fig. 4b, percentages and absolute numbers of splenic dendritic cells were increased, either in a significant or a non-significant manner, by ssRNA application in both young and aged mice (Fig. 4b). Although it was not statistically significant, the expression levels of CD25 and CD154, which are typical activation markers of T cells, seem to be increased in lung CD8^+^ T cells of young and aged mice immunized with IIV formulated with ssRNA (G3 and G6) compared with those of IIV alone immunized mice at 1 week after the boost (Fig. 4c). Moreover, the application of the ssRNA adjuvant increased the percentage of IFN-γ- and TNF-α-producing CD4^+^ T cells in the spleen of young and aged mice (G3 and G6) (Fig. 4d–g). Moreover, the numbers of IFN-γ-producing cells in the spleen and lungs were increased in both young and aged mice after HA protein stimulation (G3 and G6). Additionally, ssRNA application slightly increased the number of IL-4-producing cells in the spleen of young and aged mice (Fig. 4h). In line with these results, the culture supernatant of HA protein-stimulated splenocytes obtained from groups immunized with IIV formulated with ssRNA comprising young and aged mice (G3 and G6) showed a remarkable increase in IFN-γ and IL-2 production and a slight increase in IL-6 production (Fig. 4i).

### Alterations in mRNA expression due to ssRNA application in immunized young and aged mice

**Figure 5:**
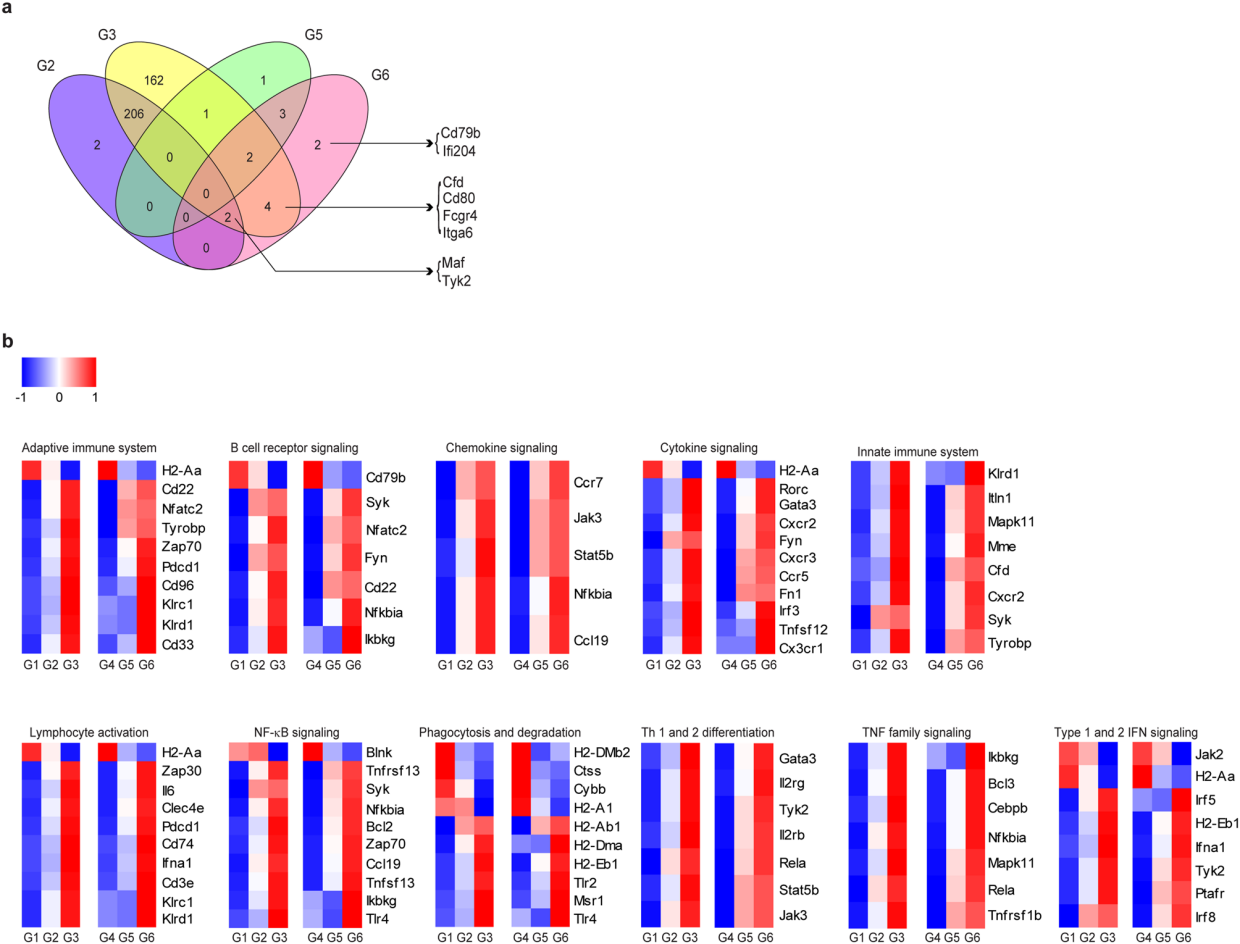
Immune-related gene expression pattern in splenocytes of immunized young and aged mice. Differentially expressed genes (DEGs) are illustrated by Venn diagrams and heatmaps. n = 3 mice for G1 to G6. (**a**) Genes whose expression levels were upregulated or downregulated more than twofold with P < 0.05 compared to G1 in young mice and G4 in aged mice are shown using Venn diagrams. (**b**) Gene expression patterns associated with immunology are shown using heatmaps.

To investigate the effects of the application of the ssRNA adjuvant on gene expression in aged mice, we compared the transcriptome profiles of splenocytes isolated from immunized mice 1 week after the boost schedule by conducting NanoString analysis. Gene expression was quantified and compared with that of the saline-immunized groups (G1 in young mice and G4 in aged mice). Differentially expressed genes (DEGs) with p < 0.05 and fold-change ≥ 2 were identified; the data are represented using a Venn diagram (Fig. 5a). We confirmed that the number of DEGs in the aged groups (G5 and G6) was lower than that in the young groups (G2 and G3); we identified 377 and 13 upregulated or downregulated genes in groups immunized with IIV formulated with ssRNA comprising young (G3) and aged (G6) mice, respectively. Among these, the expression of eight genes was upregulated [complement factor D precursor (Cfd), integrin alpha 6 (Itga6), MAF, and non-receptor tyrosine protein kinase (Tyk2)] or downregulated [cluster of differentiation 79b (Cd79b), cluster of differentiation 80 (Cd80), low-affinity immunoglobulin gamma Fc region receptor IV (Fcgr4), and interferon activable protein 204 (Ifi204)] in the groups immunized with IIV formulated with ssRNA (G6) compared with those in the IIV-immunized group (G5) in aged mice (Fig. 5a). To investigate the functional association, we analyzed gene expression patterns in the spleen of mice from each group. As shown in Fig. 5b, the ssRNA adjuvant induced several immune responses-associated gene clusters. Aged mice showed similar gene expression patterns as those in young mice. Both innate and adaptive immune system-related genes such as *Klrd1*, *ifn*α, *IL-6*, *CD74*, and *MAPK11* were upregulated by the ssRNA. Furthermore, it is expected that the ssRNA could affect the migration of immune cells and function of antigen-presenting cells as it increased several chemokine receptors and chemokines such as CCR7, CCR5, CXCR2, and Ccl19, as well as phagocytosis and type 1 and 2 IFN signaling. The ssRNA adjuvant also increased B-cell receptor signaling- (*Syk*, *Fyn*, *Nfatc2*, and *ikbkg*) and Th1 and Th2 differentiation-associated genes (*Gata3*, *IL2rg*, *Rele*, *Stat5b*, and *Jak*) (Fig. 5b and Supplementary Fig. S3).

## Discussion

Vaccination is the most effective preventive measure to tackle infectious diseases. Although vaccines are necessary for older individuals, such individuals are markedly less responsive to vaccines than younger individuals because of the occurrence of immunosenescence and inflammaging^15^. Thus, the development of novel adjuvants that can improve the efficacy of vaccines in the elderly is of considerable importance. In this study, we evaluated the adjuvant effect of ssRNA on seasonal IIV in aged and young mice. Although the application of the ssRNA adjuvant markedly increased IgG1 and IgG2a levels in aged and young mice, it exerted more prominent effects on IgG2a levels. Since the IgG2a antibody response represents a Th1 bias, it seems that ssRNA may effectively enhance the Th1 response. In fact, ssRNA application remarkably increased IFN-γ production not only in young mice but also in aged mice. Furthermore, ssRNA application considerably increased the titers of neutralizing antibodies that were not detected in the aged mice immunized with IIV alone. Thus, we suggest that ssRNA may affect the generation of follicular Th cell responses or germinal center formation because it is associated with the generation of functional neutralizing antibodies^21^. Interestingly, ssRNA application increased the BALF IgA levels even in aged mice. We believe that the increase in IgA levels confers protection against influenza virus infection in the respiratory tracts of aged mice. Thus, the use of ssRNA may widen the quantity and quality of antibody responses generated and that generally decline with aging, and it may induce not only balanced Th1/Th2 responses but also mucosal immune responses in young and aged mice.

In addition to the increase in humoral responses, ssRNA application enhanced cellular immune responses. It has been reported that effector T cells generated by aged mice express lower levels of activation and differentiation markers, such as CD154, CD25, and CD62L, and produce reduced amounts of cytokines^22^. Furthermore, a marked decrease in the helper activity of CD4^+^ T cells obtained from aged individuals was reported. Aged CD4^+^ T cells provide little cognate help to induce humoral responses, consequently leading to decreased B cell expansion, GC differentiation, and IgG production^23^. Notably, the application of our ssRNA adjuvant increased CD25 and CD154 expression in CD8^+^ cells in the lungs, as well as IFN-γ- and TNF-α-producing CD4^+^ cells in the spleen. Moreover, IFN-γ, IL-2, and IL-6 production in response to influenza protein was markedly increased in aged mice, similar to that observed in young mice. Thus, the data indicate that the ssRNA adjuvant stimulates T cell activation, and that these activated T cells might ameliorate virus-induced pathology in aged mice.

In this study, we did not determine whether ssRNA adjuvant increased the survival rate of aged mice after the challenge. However, the application of the ssRNA adjuvant alleviated the decreases in body weight, clinical illness, and BALF cytokine production as well as decreased viral titers in the lungs and BALF in both young and aged mice after the challenge. These data indicate that ssRNA application contributes to rapid virus clearance and reduction of lung inflammation^24,25^. Furthermore, the application of the ssRNA adjuvant reduced the infiltration of inflammatory cells in the lungs after the challenge, and the lungs of mice immunized with the ssRNA adjuvant and vaccine showed almost normal alveolar architecture in both young and aged mice. Therefore, we believe that ssRNA may enhance the survival rate because it effectively ameliorates influenza virus-induced pathogenesis.

In our previous study, we demonstrated that the application of the ssRNA adjuvant induced the expression of genes related to innate immune response generation and T cell activation^26^. Therefore, we examined its effect on the mRNA expression pattern of aged mice immunized with the influenza vaccine. It is known that IFN production and signaling are reduced in aged mice because of immunosenescence^6,27^. However, in the present study, the application of the ssRNA adjuvant increased Tyk2 and MAF expression in aged mice. As Tyk2 is involved in IFN signaling^28^ and MAF activates IL-4 production^29^, we anticipate that an increase in the expression of these genes may mitigate immunological defects in aged mice. Furthermore, we confirmed that the expression pattern of genes related to the innate and adaptive immune systems, cytokine signaling, lymphocyte activation, Th1 and Th2 differentiation, TNF family signaling, and type 1 and 2 IFN signaling in aged mice immunized with IIV formulated with ssRNA was similar to that observed in young mice. This indicates that ssRNA adjuvant may increase the expression of innate and adaptive immune system-related genes even in aged mice.

In conclusion, the use of the ssRNA adjuvant compensates for impaired cellular and humoral responses caused by aging, and promotes not only balanced Th1/Th2 responses but also mucosal immunity and production of neutralizing antibodies that lead to the development of protective immunity. Furthermore, its application seems to be excellent in terms of simplicity, as the formulation can be simply added to commercially available inactivated vaccines. We believe that our findings may accelerate the development of more effective influenza vaccines, particularly targeting the elderly, and may be applicable to other vaccines as well.

## Methods

### Mice

Female BALB/c mice, 6-week-old and 12-month-old, were purchased from Dae Han Bio-Link (Chungbuk, Korea). To obtain aged mice, the 12-month-old mice were housed until 21 months of age. All mice were housed in the animal facility at the Catholic University of Korea under specific pathogen-free conditions at 21–22 °C and a 12/12-h light/dark illumination cycle. All animal experimental procedures conducted in this study followed the guidelines of and were approved by the Institutional Animal Care and Use Committee of the Catholic University of Korea (approval no. CUK-IACUC-2019-004). The study was carried out in compliance with the ARRIVE guidelines.

### Vaccines and viruses

The SKYCellflu (SK Bioscience, Seoul, Korea) vaccine used against the 2018–2019 Northern hemisphere strain, is a cell culture-based quadrivalent influenza vaccine consisting of A/Michigan/45/2015(NYMC X-275), A/Singapore/INFIMH-16-0019/2016 (IVR-186), B/Phuket/3073/2013, and B/Maryland/15/2016 for A/H1N1, A/H3N2, B/Yamagata, and B/Victoria, respectively. It was used as the seasonal IIV in the present study. The A/H1N1 virus, A/California/04/2009, used in the viral infection challenges, was generously provided by Dr. Seong BL, Yonsei University, Seoul, Korea.

### In vitro transcription and RNA purification

The DNA platform was designed using the IGR IRES and SV40 late-polyadenylation signal sequences as previously described^20,26^.

DNA templates were linearized using NotI. The EZ T7 High Yield In Vitro Transcription Kit (Enzynomics, Daejeon, Korea) was used for in vitro transcription. Briefly, linearized DNA template was initially incubated with T7 transcription buffer, MgCl_2_, 10 mM DTT, Enhancer solution, 5 mM rNTP, 200 U T7 polymerase mix, and nuclease-free water in a final volume of 60 μL for 3 h at 37 °C, followed by incubation with RNase-free DNase I (Promega, Madison, WI, USA) for 15 min at 37 °C. For RNA purification, the Hi-Yield RNA Ultra-purification Kit (RBC, Banqiao, Taipei, Taiwan) was used. RNA purity and concentration were evaluated using a NanoDrop-2000 spectrophotometer (Thermo Fisher Scientific Inc., Waltham, MA, USA), and RNA integrity was analyzed by performing denaturing gel electrophoresis.

### Immunization

Young (6-week-old) and aged (21-month-old) BALB/c mice were immunized intramuscularly in the upper thigh, twice (prime and boost) at an interval of 2 weeks, with IIV only (0.6 μg) or IIV with 20 μg of ssRNA adjuvant.

### Virus challenge

The mice were challenged with 1 × 10^2^ plaque-forming units (PFU) of A/California/04/2009 (H1N1) virus (50 µL) intranasally using a pipette. After the challenge, mice body weight, survival, and clinical illness were checked. The clinical illness was scored using the following scale: 0 = no visible signs of disease; − 1 = slight ruffling of fur; − 2 = ruffled fur, reduced mobility; − 3 = ruffled fur, reduced mobility, and rapid breathing; and − 4 = ruffled fur, minimal mobility, huddled appearance, and rapid and/or labored breathing^30^. The mice were sacrificed when body weight decreased by more than 25% of the original body weight^30^.

### Serum collection

The mice were euthanized using isoflurane. Blood was collected to obtain serum samples from immunized mice at 1 or 2 weeks after the prime, boost, and virus challenge as described in the schedule (Figs. 1a, 4a). Blood samples were collected from the facial vein using an 18-G needle or from the abdominal vena cava using a 1-mL syringe; samples were subsequently microcentrifuged for 15 min at 7000 rpm and 13,000 rpm, respectively. The sera were stored at − 80 °C until use.

### Bronchoalveolar lavage (BAL) fluid collection

BAL of the sacrificed mice was performed using a 22-gauge catheter and 1 mL of saline by flushing the airway compartment three times. The BALF was centrifuged at 13,000 rpm for 10 min at 4 °C.

### Enzyme-linked immunosorbent assay (ELISA)

ELISAs were conducted to measure Antigen-specific IgG1 and IgG2a levels in mouse serum and antigen-specific IgA levels in mouse BALF as described in pervious report^19^. The 96-well plates (Corning, Corning, NY, USA) were coated with IIV (100 ng/well) for 2 h at 18–25 °C. After 2 h incubation, the wells were washed three times with 200 µL of PBS-T (phosphate-buffered saline [PBS] containing 0.05% Tween 20) and blocked with 100 µL of blocking buffer (PBS containing 1% bovine serum albumin) for 1 h at 18–25 °C. Serum samples were diluted 1/200–1/400 in blocking buffer, added to the wells, and incubated overnight at 4 °C. After incubation, the wells were washed three times with 200 µL of PBS-T. Anti-mouse IgG1- (Invitrogen, Carlsbad, CA, USA), IgG2a- (Novus Biologicals, Centennial, CO, USA), and IgA-HRP- (Bethyl Laboratories, Inc., Montgomery, TX, USA) conjugated antibodies were diluted 1/1000–1/5000 using blocking buffer and incubated for 1 h at 18–25 °C. After five washes with PBS-T, tetramethylbenzidine substrate (Invitrogen) was added, and the samples were incubated for 5–10 min; then 2 N H_2_SO_4_ was added to terminate the reaction. The optical density (OD) values were measured at 450 nm using a GloMax Explorer microplate reader (Promega).

To measure the levels of cytokines in the splenocyte culture supernatants, splenocytes were isolated from immunized mice, seeded at 5 × 10^5^ cells/well in a 96-well plate, and stimulated with 500 ng/well of IIV for 72 h at 37 °C. The concentrations of IFN-γ, IL-2, and IL-6 were determined using ELISA kits (Invitrogen; Thermo Fisher Scientific Inc.), according to the manufacturer’s instructions. The concentrations of these cytokines were calculated using the respective standard curves, and the results obtained are shown as the amount (pg) of each cytokine per milliliter of supernatant.

### Hemagglutination inhibition assay

The hemagglutination inhibition assay was performed according to the “Manual for the laboratory diagnosis and virological surveillance of influenza” (https://www.who.int/influenza/gisrs_laboratory/manual_diagnosis_surveillance_influenza/en/). Briefly, mouse sera were treated with receptor-destroying enzyme (Denka Seiken, Tokyo, Japan) overnight at 37 °C and then inactivated for 30 min at 56 °C, followed by adsorption with chicken red blood cells to prevent elicitation of nonspecific responses. All sera were then twofold serially diluted in 25 μL of PBS in V-bottom 96-well microtiter plates and incubated with standardized viral suspensions (4 HA U/25 μL) at 18–25 °C for 1 h. Fifty-microliters volumes of 0.5% chicken RBCs were added, and the plates were incubated for 45 min at 18 ~ 25 °C. The antibody titers (geometric mean titers, GMT) were expressed as the reciprocal of the highest serum dilution that showed complete inhibition of agglutination in duplicate experiments. As the initial dilution was 1/10, the lower limit of the detectable antibody titer was 1:10. Titers < 1:10 were assigned a value of 1:5 for calculation purposes.

### Microneutralization (MN) assay

Serum samples were inactivated for 30 min at 56 °C. All serum samples were then twofold serially diluted starting from 1:10 in serum-free medium in 96-well cell culture plates (Corning) and incubated with the same volume of viral suspension containing 25 TCID_50_ of influenza A/California/04/2009 (H1N1) at 37 °C for 1 h in a humidified atmosphere with 5% CO_2_. After incubation, 100 μL of the mixture at each dilution was added to a cell plate containing a Madin–Darby Canine Kidney (MDCK) monolayer. The plates were incubated for 48 h at 37 °C in a humidified atmosphere with 5% CO_2_, after which the supernatant was carefully discarded and 100 μL of a 5% formaldehyde solution (DaeJung Chemicals & Metals, Siheung-si, Gyeonggi-do, Korea) was added. After 30 min incubation at room temperature, the 5% formaldehyde solution was discarded and 100 μL of 0.1% crystal violet solution (Sigma-Aldrich, St, Louis, MO, USA) was added. After 1 h of incubation, the crystal violet solution was discarded, and the cell monolayer was washed using tap water. The results were evaluated using a microscope. The highest protective serum dilution was considered as the neutralization titer^31^.

### Real-time PCR for virus titration

Virus titers were quantified by real-time PCR methods as previously reported^19,20^. Total RNA was isolated from the lungs and BALF using the TRIzol reagent (Favorgen, Ping-Tung, Taiwan). The PCR reaction contained 10 μL of template RNA, standard, or negative control; 12.5 μL of 2 × SuperScript III Platinum Master Mix (Invitrogen); 0.5 μL of SuperScript III Taq polymerase (Invitrogen); and 2 μL each of forward primer (10 μM), reverse primer (10 μM), and dual-labeled probe (5 pmol) in a total volume of 25 μL. Real-time PCR was performed using a Bio-Rad thermocycler (Bio-Rad Laboratories Inc., Hercules, CA, USA). The PCR conditions were as follows: 30 min at 50 °C and 5 min at 95 °C, followed by 45 cycles of 20 s at 95 °C and 1 min at 55 °C. For virus detection, we used two pairs of influenza virus-specific primers and TaqMan probes designed based on the conserved matrix gene region of influenza A virus. The sequences included: forward primer 5′-GACCRATCCTGTCACCTCTGAC-3′, reverse primer 5′-AGGGACTTYTGGACAAAKCGTCTA-3′, and probe 5′-FAM-TGCAGTCCTCGCTCACTGGGCACG-BHQ1-3′.

### Histological analysis

The lung tissue samples obtained from experimental mice were submerged in 10% formalin for 48 h, paraffin-embedded, and sectioned to 3–5 μm thickness before mounting and staining for routine hematoxylin and eosin (H&E)-based analysis. Representative histopathological images were obtained and evaluated using Aperio ImageScope Version 12.3 (Leica Biosystems, Buffalo Grove, IL, USA). The histopathologist was blinded to the group distribution.

### Cytokine analysis in BALF samples

The concentrations of IL-4, IL-6, IL-10, IFN-γ, and TNF-α in BALF samples were determined using the Magnetic Luminex Screening Assay Kit (R&D Systems, Inc., Minneapolis, MN, USA) according to the manufacturer’s instructions.

### Flow cytometry

To determine the HA tetramer, the isolated splenocytes and lung lymphocytes were blocked using CD16/CD32 (eBioscience; Thermo Fisher Scientific Inc.) and 5 μg streptavidin (Invitrogen) for 20 min at 4 °C, and then stained using CD8a (clone 53-6.7, eBioscience), H-2Kd HA tetramer with the HA518-526 epitope IYSTVASSL, CD4 (clone GK1.5, eBioscience), CD45 (clone 30-F11, eBioscience), and Fixable Viability Dye eFluor520 (eBioscience) for 30 min at 4 °C in the dark. The HA tetramer was produced and generously provided by Dr. Chang Jun, Ewha Womans University, Seoul, Korea. The stained cells were fixed using 4% paraformaldehyde. To determine macrophage, dendritic cell, and T cell activation, isolated splenocytes and lung lymphocytes were blocked using CD16/CD32 (eBioscience) for 20 min at 4 °C and stained using F4/80 (clone BM8, eBioscience), CD11c (clone N418, eBioscience), CD8a (clone 53-6.7, eBioscience), CD25 (clone PC61.5, eBioscience), CD154 (clone MR1, eBioscience), CD45 (clone 30-F11, eBioscience), and Fixable Viability Dye eFluor520 (eBioscience) for 30 min at 4 °C in the dark. Stained cells were fixed using 4% paraformaldehyde. To evaluate the generation of cytotoxic T cell responses, isolated splenocytes were stimulated with 500 ng/well IIV for 16 h at 37 °C and then Bredfeldin A (GolgiPlug, BD Biosciences, Franklin Lakes, NJ, USA) was added after 6 h. After another 10 h of incubation, splenocytes were blocked with CD16/CD32 (eBioscience) for 20 min at 4 °C and first stained with CD4 (clone GK1.5, eBioscience) and Fixable Viability Dye eFluor520 (eBioscience) for 30 min at 4 °C in the dark. The stained cells were permeabilized using a Fixation/Permeabilization Solution kit (BD Biosciences) for 1 h at 4 °C in the dark, and then stained with IFN-γ (clone XMG1.2; eBioscience) and TNF-α (clone MP6-XT22; eBioscience) for 30 min at 4 °C in the dark. Cells were analyzed using the FACS Aurora (Cytek Biosciences, Fremont, CA, USA), and the data were analyzed using Spectroflo (Cytek Biosciences) or FlowJo (Tree Star, Inc., Ashland, OR, USA).

### Enzyme-linked immunospot (ELISPOT) assay

Splenocytes and lung lymphocytes were stimulated with 500 ng/well IIV or 5 μg/well HA-specific T cell epitope peptide mixture (LYEKVRNQL, GIPSRISI, GMVDGWYG, VRPVTSGCF, RGFFGAIAGF, and IYSTVASSL) for 48 h at 37 °C. HA peptides were synthesized by Peptron (Daejeon, Korea). The ELISPOT assays for the detection of IFN-γ or IL-4-secreting T cells were conducted using mouse IFN-γ and IL-4 ELISpotBASIC (Mabtech, Stockholm, Sweden). Each step was performed according to the manufacturer’s instructions.

### NanoString-based analysis

Spleen samples from immunized mice were obtained 7 days after the boost schedule. Total RNA was isolated from spleen samples using the TRIzol reagent and confirmed using an Agilent RNA 6000 Pico kit (Agilent Technologies, Santa Clara, CA, USA) and a bioanalyzer. Total RNA was analyzed using the NanoString nCounter Analysis System (NanoString Technologies, Inc., WA, USA) according to the manufacturer's instructions. A 5 μL (100–300 ng) aliquot of each RNA sample was mixed with 8 μL of the Master Mix (reporter codeSet and hybridization buffer), 2 μL of the capture probeSet was added, and the sample was placed in a 65 °C thermocycler (Bio-Rad Laboratories Inc.) for 16 h. The samples were then transferred to the preparation station (NanoString Technologies) with a prepared nCounter Master Kit and a cartridge. After binding the sample to the cartridge, 12 lanes per run of the nCounter prep station were performed for approximately 2.5–3 h. The cartridges were transferred to a Digital Analyzer (NanoString Technologies) for analysis and scanned at 555 fields of view. To calculate gene expression values, the first background correction used default options with control probes in Codeset. Next, data normalization also used control probes in Codeset. The scaling of data was controlled by using the expression level of genes overlapping between each code set. Finally, DEGs between the two selected biological conditions were analyzed by performing the Student’s *t*-test. All processes, such as background correction and normalization as well as statistical calculations were performed using nSolver Analysis software ver 4.0. (NanoString).

### Statistical analysis

Analyses were performed using a one-way ANOVA with Tukey’s post hoc test for multiple-group comparisons and unpaired *t*-tests to compare between two groups. Differences were considered significant at p < 0.05. Data are expressed as mean ± SD. Statistical analyses were conducted using GraphPad Prism 8 (GraphPad Software Inc., San Diego, CA, USA) and SPSS for Windows (release 14.0 K, SPSS Inc., IL, Chicago, USA).

## Supplementary Information

Supplementary Figures.

## Data Availability

All substantial data are available in the text and the Supplementary Figures. Certificates of analysis and origins, material data sheets, and detailed procedures are available upon request to the corresponding author.
