# Supplementary material for: Effective inactivated influenza vaccine for the elderly using a single-stranded RNA-based adjuvant
Source: Sci Rep. 2021 Jun 7;11:11981. doi: 10.1038/s41598-021-91445-3 (PMC8184738; doi:10.1038/s41598-021-91445-3)
Supplement: Supplementary file 1 — Supplementary Figures. [file 41598_2021_91445_MOESM1_ESM.docx]

**Effective inactivated influenza vaccine for the elderly using a single-stranded RNA-based adjuvant**

Yoo-Jin Bang^1,2,^ ^¶^, So-Hee Hong^1,2,^ ^¶^, Hyo-Jung Park^1,2,^ ^¶^, Hye Won Kwak^1,2^, Yu-Sun Lee^1,2^, Jae-Yong Kim^1,2^, Hyeong-Jun Park^1,2^, Seo-Hyeon Bae^1,2^, Hye-Jung Kim^1^, Yun-Hee Kim^3^, Hae Li Ko^4^, Sang-In Park^4^, Hun Kim^3^, Gyeongjoo Park^5^, Man-Seong Park^6^, Jun Chang^5^, Jae-Hwan Nam^1,2,^ *

^1^ Department of Medical and Biological Sciences, The Catholic University of Korea, Gyeonggi-do, Bucheon, Republic of Korea

^2^ BK Plus Department of Biotechnology, The Catholic University of Korea, Gyeonggi-do, Bucheon, Republic of Korea

^3^ Department of R&D, SK bioscience, Pangyoro, Bundang-gu, Republic of Korea

^4^ Scripps Korea Antibody Institute, Chuncheon, Kangwon-Do, Republic of Korea

^5^ Graduate School of Pharmaceutical Sciences, Ewha Womans University, Seoul, Republic of Korea

^6^ Department of Microbiology, College of Medicine, Korea University, Seoul, Republic of Korea

**
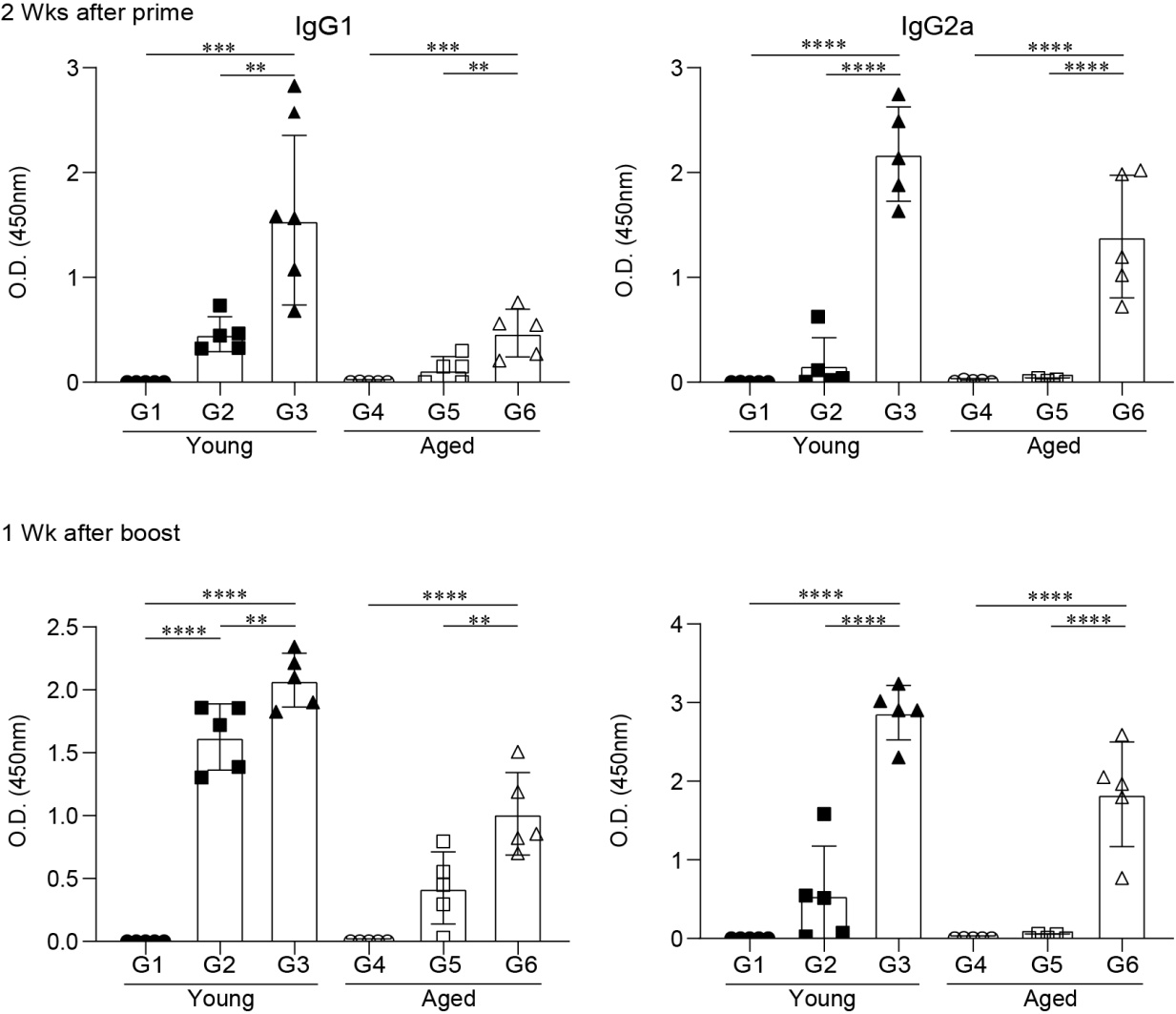
**

**Supplementary Figure S1.** **Inactivated influenza vaccine (IIV) formulated with ssRNA improves IIV-induced humoral responses.** BALB/c mice were intramuscularly immunized with 0.6 μg IIV with or without ssRNA adjuvant at an interval of 2 weeks. For IgG1 and IgG2a measurement, sera were collected two weeks after the prime and one week after the boost schedule. Data are represented as mean ± SD. The data were statistically analyzed using one-way ANOVA. The significance of differences between the groups is indicated with bars and symbols as follows: **, p < 0.01; ***, p < 0.001; ****, p < 0.0001. n = 5 mice for G1 to G6. IIV-specific IgG1 and IgG2a levels were measured by performing ELISA two weeks after the prime and one week after the boost schedule.

**
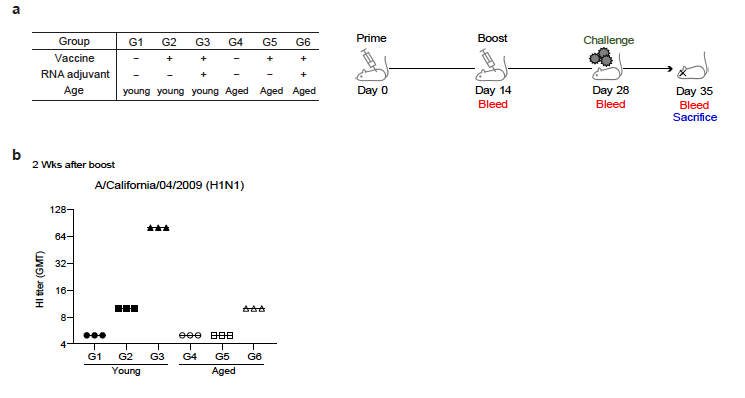
**

**Supplementary Figure S2. ssRNA formulated inactivated influenza vaccine (IIV) increased HI titers against A/California/04/2009 (H1N1) in both young and old mice.**

(**a**) Overall study design. BALB/c mice were intramuscularly immunized with 0.6 μg IIV with or without ssRNA adjuvant at an interval of two weeks. The mice were challenged with A/California/04/2009 virus two weeks after the boost schedule.

(**b**) Hemagglutination inhibition titer against vaccine strains measured by hemagglutination inhibition assay two weeks after the boost schedule.

**
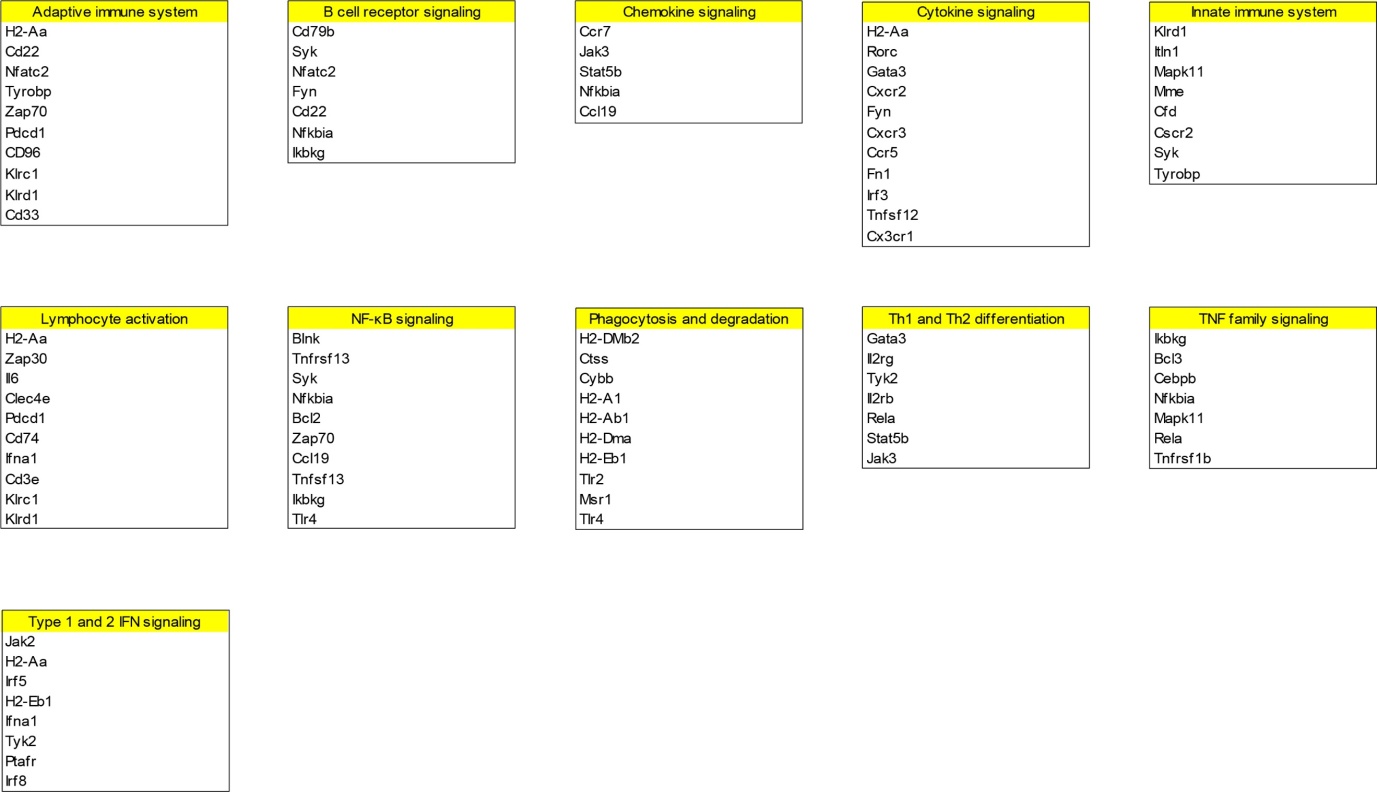
**

**Supplementary Figure S3. Comparison of gene expression patterns.** Genes associated with immunology are shown.
